# The Effects of COVID-19 Vaccine Mandates in Hawaii

**DOI:** 10.3390/vaccines10050773

**Published:** 2022-05-13

**Authors:** Ruben Juarez, Nicole Siegal, Alika K. Maunakea

**Affiliations:** 1 Economics and UHERO, University of Hawaii—Mānoa, Honolulu, HI 96822, USA; nsiegal@hawaii.edu; 2 John A. Burns School of Medicine, University of Hawaii, Honolulu, HI 96813, USA; amaunake@hawaii.edu

**Keywords:** COVID-19 vaccine, mandates, business impacts, Hawaii

## Abstract

Having been affected by the highest increase in COVID-19 cases since the start of the pandemic, Honolulu and Maui counties in Hawaii implemented vaccine passport mandates for select industries in September 2021. However, the degree to which such mandates impacted COVID-19 mitigation efforts and economics remains poorly understood. Herein, we describe the effects of these mandates on changes in three areas using difference-in-difference regression models: (1) business foot traffic; (2) number of COVID-19 cases per 100,000 individuals, and (3) COVID-19 vaccination rates across counties affected or unaffected by the mandates. We observed that although businesses affected by mandates experienced a 6.7% decrease in foot traffic over the 14 weeks after the mandates were implemented, the number of COVID-19 cases decreased by 19.0%. Notably, the vaccination rate increased by 1.41% in counties that implemented mandates. In addition, towards the end of the studied period, the level of foot traffic at impacted businesses converged towards the level of that of non-impacted businesses. As such, the trade-off in temporary losses at businesses was met with significant gains in public health and safety.

## 1. Introduction

Faced with the largest increase in cases since the beginning of the COVID-19 pandemic, on 13 September 2021, the counties of Honolulu and Maui in the state of Hawaii mandated that customers and employees of establishments in the food and beverage, fitness, and entertainment industries provide proof of full vaccination or a recent negative COVID-19 test [1]. This is sometimes referred to as a “vaccine or test passport.” Some business owners did not approve of the measure, fearing further losses like those seen at the beginning of the pandemic [2], when there was a 22% decline in the number of active small businesses in the U.S. from February to April of 2020 [3]. Reports from previous safety mandates have demonstrated the decreased spread of COVID-19, such as in states with face mask mandates that observed a 2.0% decrease in cases over 21 days after implementation from March to May 2020 [4]. However, the impacts of other public health mandates, such as for COVID-19 vaccines, has yet to be reported.

To evaluate the degree to which vaccine mandates impacted businesses and COVID-19 mitigation efforts, we analyzed data in Hawaii as it was the first state in the U.S. to implement vaccine mandates during the pandemic. Specifically, we evaluated the impacts on the 7-day average daily foot traffic from a panel of nearly 20,000 businesses, the 7-day average daily new COVID-19 cases per 100,000 individuals, and the weekly vaccination rate increases in the state using difference-in-difference regression models. Using a novel dataset from foot-traffic, vaccination, and COVID-19 testing data, we found that businesses that required employees and customers to be vaccinated (or regularly tested for COVID-19) experienced a significant decrease in foot traffic (p<0.01, 95% Cl: 5.7–6.9%) compared to those not required in the 14 weeks following the implementation of the vaccine mandate. We also found that the counties that implemented this mandate (Honolulu and Maui counties) experienced a significantly lower rate of new COVID-19 cases per 100,000 individuals (p<0.01, 95% Cl: 12.1–25.8%) than those that did not implement the same measures (Kauai and Hawaii counties). Finally, we observed that counties with the mandates had a significantly higher rate in the weekly increases of the adult vaccination rate (p<0.05, 95% Cl: 0.29–2.55%) compared to counties without a mandate. These results held when including controls for flight passenger arrivals and fixed effects for businesses, counties, days, and day-of-the-week.

In part because of the COVID-19 vaccine mandate, 96% of adults in Hawaii have been vaccinated as of 1 December 2021, surpassing every state in the U.S. [5]. Studies estimate that control of COVID-19 might be achieved when 60–70% of the world’s population is vaccinated against it [6,7]. Although some businesses suffered an initial loss due to decreased numbers of customers in the early stage of the mandates, the increased safety of individuals and promotion of public health later allowed them to return to normal operations. As the trend for COVID-19 cases decreased and vaccination of adults increased, Hawaii counties allowed businesses under these mandates to loosen capacity restrictions [1]. Results of our study show that the benefits of the vaccine mandates to public health safety and long-term economic recovery far outweighed the acute disruptions to Hawaii’s businesses.

## 2. Materials and Methods

### 2.1. Data Sources

The data for this study was compiled from five sources. The foot-traffic data was provided by SafeGraph. The data was created from a panel of 20 million devices that collect anonymous location data. All device users gave permission for a variety of mobile apps to track their location. This report uses data for visits to 19,625 businesses in the state of Hawaii. SafeGraph tracks the number of times a member of their panel enters one of these businesses each day. This data was primarily targeted for use in firms to analyze retail traffic. They also share data with academic researchers. As stated on their website, “SafeGraph, a data company that aggregates anonymized location data from numerous applications in order to provide insights about physical places, via the SafeGraph Community. To enhance privacy, SafeGraph excludes census block group information if fewer than five devices visited an establishment in a month from a given census block group” [8]. Other researchers have used this data to analyze social distancing during the pandemic, including studies on how inequality contributes to individuals’ ability to social distance [9]. Another study used this data to track movement following the Sturgis motorcycle rally in August 2020 on the resulting spread of COVID-19 [10].

We matched this data by day and county with COVID-19 positive case data provided by the State of Hawaii Department of Health’s Disease Outbreak Control Division [11]. They provide the number of COVID-19 test encounters and the number of positive cases. The population data is from the United States Census Bureau estimates from the 2020 Census [12]. We calculated the new cases per 100,000 individuals by rolling the 7-day average for each county by dividing the 7-day average of new positive tests by the population over 18 for each county in each day and multiplying by 100,000, using the following formula:

(1) $$Cases\;Per\;{100,000}_{cd}=\frac{7\text{-}day\;Average\;New\;Cases_{cd}}{over\;18\;population_{c}}*100,000$$

The weekly vaccination data was provided by the State of Hawaii, Department of Health (DOH) Disease Outbreak Control Division [11]. This data is unpublished DOH data derived from the Hawaii Immunization Registry (HIR). These numbers include vaccines administered in the State of Hawaii reported to the Vaccine Administration Management System and transferred to HIR, as well as doses reported by providers directly to HIR. Some federal agencies have not reported administered doses to either system, so most federal agency doses are not reflected in these counts. These counts are the most accurate and reliable currently available in the state. The data was provided by week, county, age group, and dose (1, 2, or 3). We focused on initial doses taken by adults. The weekly vaccination rate increase was measured by the number of adults who received their first COVID-19 vaccine divided by the over-18 population for each county and multiplied by 100, as shown in the following formula:

(2) $$Vaccination\;Rate\;Increase_{cw}=\frac{New\;Vaccinations_{cw}}{over\;18\;population_{c}}*100$$

Finally, this data was matched again by day and county with the number of airline passenger arrivals. The State of Hawaii’s Department of Business, Economic Development & Tourism provides visitor statistics on the Daily Passenger Count Dashboard [13]. As many businesses and employees in the state depend on tourism; this value was expected to impact both foot traffic and COVID-19 case rates.

### 2.2. Empirical Strategy

**Figure 1:**
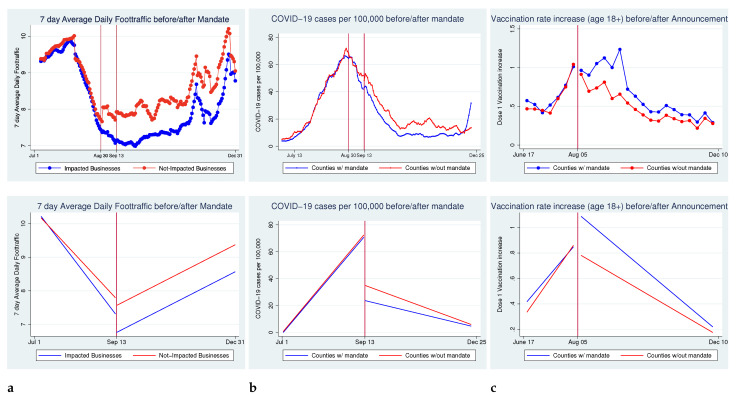
Pre- and post- mandates values and fitted trends for impacted and non-impacted businesses or counties. (**a**) 7-day rolling average foot traffic before and after implementation of the mandates, by businesses impacted or not. (**b**) 7-day rolling average of new COVID-19 cases per 100,000 individuals before and after implementation of the mandates, by counties with or without mandates. (**c**) Increase in county vaccination rates of the first dose administered weekly for all adults before and after the announcement of the mandates, by counties with or without mandates.

To determine the impact of the COVID-19 vaccine or test passport mandates on foot traffic to impacted businesses, COVID-19 cases per 100,000 individuals, and the vaccination rate in counties with mandates, we use difference-in-difference regressions. The difference-in-difference estimation model calculates the difference in the average outcome before and after treatment in the treatment group minus this difference in the outcome in the control group before and after treatment- thus, the name “difference-in-differences” [14]. The primary assumption of this model was that the treatment and control groups followed the same trend prior to the treatment, called the “parallel trends assumption” [14]. As shown in Figure 1, the treatment and control counties do follow the same trend prior to treatment for all variables. Some limitations of this model are provided in the discussion section.

#### 2.2.1. Foot Traffic Model

The following model was used to evaluate the mandate’s impact on foot traffic from July through December of 2021, with the mandates beginning on 13 September 2021:

(3) $$\begin{array}{c} ln\left(FootTraffic_{idc}\right)=\beta_{0}+\beta_{1}t_{d}+\beta_{2}Impacted_{i}+\beta_{3}t_{d}*Impacted_{i}+\\ \beta_{4}ln\left(CovidCases_{cd}\right)+\beta_{5}ln\left(Arrivals_{cd}\right)+\gamma_{c}+\gamma_{weekday}+e_{idc} \end{array}$$

and when including individual business fixed effects:

(4) $$\begin{array}{c} ln\left(FootTraffic_{idc}\right)=\alpha_{0}+\alpha_{1}t_{d}+\alpha_{2}t_{d}*Impacted_{i}+\alpha_{3}ln\left(CovidCases_{cd}\right)+\alpha_{4}ln\left(Arrivals_{cd}\right)+\gamma_{weekday}+\gamma_{i}+e_{idc} \end{array}$$

where $FootTraffic_{idc}$ represents the number of visitors to business *i* on day *d* in county *c*. $t_{d}$ is equal to one on days following the mandates going into effect (13 September 2021 and after) and equal to zero prior. $Impacted_{i}$ is equal to one for businesses that fall into the impacted categories, including food, beverage, entertainment, fitness, and the arts. $CovidCases_{cd}$ and $Arrivals_{cd}$ represent the new COVID-19 cases and passenger arrivals in county *c* on *d*. $\gamma_{c}$, $\gamma_{weekday}$, and $\gamma_{i}$ represent fixed effects for the county, day-of-the-week, and individual business, respectively. When including individual business fixed effects, the county and impact status become redundant.

The coefficient on the interaction of post-treatment and impacted business, $\beta_{3}$ and $\alpha_{2}$ represent the difference-in-differences coefficients. These represent the change in the trend between impacted businesses and non-impacted businesses following the implementation of the mandates, making this our variable of interest.

#### 2.2.2. COVID-19 Cases Per 100,000 Individuals

We use a similar model to determine the mandate’s impact on COVID-19 cases per 100,000 individuals in counties that did and did not implement the mandates. As more recent data was available and to create a balanced panel, data from 1 July 2021 through 20 December 2021 was used in the following regression:

(5) $$\begin{array}{c} Cases\;Per\;{100,000}_{cd}=\delta_{0}+\delta_{1}t_{d}+\delta_{2}HM_{c}+\delta_{3}t_{d}*HM_{c}+\delta_{5}ln\left(Arrivals_{cd}\right)+\gamma_{c}+\gamma_{weekday}+e_{cd} \end{array}$$

and when including daily fixed effects:

(6) $$Cases\;Per\;100,000cd=\lambda_{0}+\lambda_{1}HM_{c}+\lambda_{2}t_{d}*HM_{c}+\lambda_{5}ln\left(Arrivals_{cd}\right)+\gamma_{c}+\gamma_{d}+e_{cd}$$

where $t_{d}$, $ln(Arrivals_{cd})$, $\gamma_{c}$ and $\gamma_{weekday}$ have the same meaning as the prior model. In addition, the variable $CasesPer{100,000}_{cd}$ represents the rolling 7-day average COVID-19 cases per 100,000 individuals in county *c* on day *d*. $HM_{c}$ is equal to one in counties that have a mandate (Honolulu and Maui) and equal to zero in those without (Kauai and Hawaii). $\gamma_{d}$ represents daily fixed effects. Again, the interaction of post-mandate and impacted observations will provide us the difference-in-differences estimate, coefficients $\delta_{3}$ and $\lambda_{2}$.

#### 2.2.3. Vaccination Rate Increases

A third difference-in-difference model was deployed to estimate the impact of the mandates on the weekly vaccination rate increases in counties with and without the mandates. As many individuals needed to be vaccinated by the start of the mandates to maintain their current employment or frequent certain establishments, the main impact on vaccination is seen after the announcement of the mandates on 5 August 2021. We estimated this model weekly from 17 June 2021, to 11 October 2021. The following model was used:

(7) $$\begin{array}{c} Vaccination\;Rate\;Increase_{cw}=\zeta_{0}+\zeta_{1}announce_{w}+\zeta_{2}HM_{c}+\zeta_{3}announce_{w}*HM_{c}+\\ \zeta_{4}PosRate_{w}+\zeta_{5}ln\left(Arrivals_{cw}\right)+\gamma_{c}+e_{cw} \end{array}$$

and when including week fixed effects:

(8) $$\begin{array}{c} Vaccination\;Rate\;Increase_{cw}=\rho_{0}+\rho_{1}HM_{c}+\rho_{2}announce_{w}*HM_{c}+\rho_{3}PosRate_{w}+\\ \rho_{4}ln\left(Arrivals_{cw}\right)+\gamma_{c}+\gamma_{w}+e_{cw} \end{array}$$

where $ln(Arrivals_{cd})$, $HM_{c}$$\gamma_{c}$ have the same meaning as the prior models. In addition, the variable $Vaccination\;Rate\;Increase_{cw}$ is the increase in the vaccination rate in county *c* in week *w*. $announce_{w}$ is equal to one following the announcement of the mandates (5 August 2021 and after) and zero prior. $\gamma_{w}$ represents weekly fixed effects. The coefficients of interest, $\zeta_{3}$ and $\rho_{2}$, represent the difference-in-difference interaction to estimate the difference in trends between counties with and without mandates, following the announcement.

## 3. Results

### 3.1. Noticeable Trends

The state requirement imposed on employees and customers to be either vaccinated or subjected to regular COVID-19 testing, hitherto referred to as the “vaccine mandate”, was announced on 5 August 2021, officially proclaimed on 30 August 2021, and implemented on 13 September 2021 for businesses in the food, beverage, entertainment, fitness and arts industries located in Honolulu and Maui counties. Businesses located in other counties (Hawaii and Kauai) were not subjected to this mandate. To measure the impact of this mandate on businesses across various sectors, we first focused on daily foot traffic data obtained in collaboration with SafeGraph from 1 July to 30 December 2021, summarized in Table 1 and discussed in detail in the Methods section. Of 19,625 individual businesses included in this evaluation, 28.70% were subjected to the vaccine mandate; data from the remaining 71.30% that were not subjected to the mandate were used for comparison. The primary outcome variable measured relevant to businesses is the daily average foot traffic. In addition, two additional variables measured relevant to public health are the 7-day rolling average of new COVID-19 cases per 100,000 individuals and the increase in county vaccination rates of the first dose administered weekly for all adults.

In Figure 1, we show the trends for all three of the outcome variables before and after the mandates or announcement of the mandates. In Figure 1a, the rolling 7-day average daily foot traffic for businesses impacted and not impacted by the mandates were quite similar prior to the official proclamation and implementation. However, once the mandates took effect, there was a lower level of foot traffic among impacted businesses. Towards the end of the 14 weeks following implementation, we observed that impacted businesses had increased foot traffic, returning to levels similar to those found among non-impacted businesses. Figure 1b shows the COVID-19 cases per 100,000 individuals in counties with and without mandates. Prior to implementation, all counties followed a similar trend, including throughout the Delta-driven COVID-19 surge just before the mandates began. Once the mandates were in effect, impacted counties had a notably lower level of cases per 100,000 individuals. Lastly, in Figure 1c, we observed that counties with the mandates had a higher weekly vaccination increase throughout the period, with a significant increase following the announcement of the mandates, as many individuals prepared for the requirement. In order to capture this uptake, we evaluate vaccinations in later regressions from 17 June 2021 to 11 October 2021.

The mandates’ impacts on the trend of foot traffic can be seen in Figure 1a. Prior to the mandates, both impacted and not impacted businesses saw similar trends in decreasing foot traffic, most likely due to confusion surrounding the start of the requirements and the decreasing number of tourists as summer was ending. In our regressions, we control for the tourism levels, as well as COVID-19 spikes in cases that may also have impacted the number of visits to businesses. Following the implementation of the mandates, all businesses showed a slow upward trend, with impacted businesses at a lower level of foot traffic than non-impacted businesses. This leads us to believe that the mandates initially decreased visits to businesses following its implementation.

Although businesses are shown trending back towards normal levels as more people became accustomed to the mandates and got vaccinated, the impact on the spread of the virus shows the necessity of such a policy, as seen in Figure 1b. Prior to the mandates, the state was experiencing an increase in cases due to both the Delta variant and increased tourism in the summer months for all counties, with the counties that would implement mandates having a very similar number of cases per 100,000 individuals compared to those which did not. However, once the mandates were in effect, the counties with a mandate showed a lower number of cases per 100,000 individuals than the counties without mandates.

One of the direct goals of the mandates was to increase vaccine uptake in these counties. Prior to the announcement, the weekly increases in the vaccination rate were fairly similar across counties, as seen in Figure 1c. However, following the announcement of the mandates on 5 August 2021, many individuals chose to become vaccinated. We focused on adults (age 18 or older) in this study as the mandates’ target population was working-age adults and those that frequent bars, restaurants, and gyms.

### 3.2. Regression Results

#### 3.2.1. Impacts of COVID-19 Vaccine or Test Passport Mandates on Foot Traffic

Columns (1)–(4) of Table 2 show the results of the difference-in-differences models described in Equations (3) and (4). Across specifications, the difference-in-difference coefficient on the interaction shows that the businesses that fell under the mandates had a significantly (6.7%, p<0.01, 95% Cl: 5.7–6.9%) lower amount of foot traffic compared to the trend seen for non-impacted businesses during this period. This holds when including individual business, county, and day-of-the-week fixed effects. In column (4), which includes all fixed effects, the significant (p<0.01, 95% Cl: 0.057–0.069%) coefficient of −0.0672 represents a 6.7% decrease from the trend seen in non-impacted businesses, or with an average of 8.5 visits per day, an average of 0.57 fewer daily visits for impacted businesses.

The other coefficients provide a consistent picture of these trends. There was a small decrease in visits after the mandates went into effect, which may in part be due to some impacted businesses switching from dine-in to takeout and delivery only. Businesses that fell under the mandates had higher daily visits. As the impacted businesses included restaurants, bars, and gyms; they were expected to have many visitors. The daily COVID-19 cases in each county decreased the number of visitors, as individuals may venture out less if they are avoiding infection. Lastly, as expected, the number of flight passenger arrivals in each county increased foot traffic as there were more people, particularly tourists, visiting the various businesses.

#### 3.2.2. Impacts of COVID-19 Vaccine or Test Passport Mandates on COVID-19 Cases per 100,000 Individuals

Columns (5)–(7) of Table 2 show the results of the difference-in-difference models describing the impacts on the rolling 7-day average COVID-19 cases per 100,000 individuals, as described in Equations (5) and (6). In each regression, the interaction of counties with mandates and when the mandates were in effect, the difference-in-difference coefficient, was negative and significant (p<0.01, 95% Cl: 4.2–14.6%. When including day fixed effects, the post-implementation period saw counties with mandates having 6.6 fewer cases per 100,000 individuals than counties without a mandate. When instead of using day-of-the-week fixed effects, this impact was slightly higher at 9.9 fewer cases per 100,000 individuals. For reference, this is 19.0–28.4% (p<0.01, 95% Cl: 12.1–41.7%) of the daily average prior to the mandates of 35 cases per 100,000 individuals.

#### 3.2.3. Impacts of COVID-19 Vaccine or Test Passport Mandates on Weekly COVID-19 Vaccination Rate Increases

Columns (8)–(13) of Table 2 show the estimates for the difference-in-difference models in Equations (7) and (8). In each regression, we observe that the trend of the weekly vaccination rate increase was positive and significantly (p<0.05, 95% Cl: 0.031–0.283%) higher in counties with mandates following the announcement than in those without mandates. When including weekly fixed effects, the coefficient of 0.157 (p<0.05, 95% Cl: 0.031–0.283%) on the interaction indicates the average vaccination rate increase per week in counties with a mandate in comparison to those without a mandate. This added to a total of 1.41 (p<0.05, 95% Cl: 0.29–2.55%) percentage points higher vaccination rate over the 9 weeks following the announcement.

## 4. Discussion

Mandatory vaccination has been highly controversial across the world. Although mandatory vaccination programs have proven effective in increasing vaccine uptake in Denmark, France, Germany, Israel, Italy, and Switzerland [15], Canada [16], and Australia [17], understanding the complexity of mandates in the United States has been difficult due to the heterogeneity of policies across different states. Furthermore, the impact of mandates on businesses adopting the mandates has not been evaluated.

We observed strong evidence that the vaccine or test passport mandates implemented in Honolulu and Maui counties in Hawaii impacted businesses and public health by decreasing daily foot traffic to impacted businesses and decreasing COVID-19 cases per 100,000 individuals and increasing weekly vaccination rates in the impacted counties. While the initial impact on businesses, many of whom struggled throughout the pandemic, may have caused initial losses, the gains to public health through decreasing COVID-19 cases and increasing vaccination, as shown in this study, may provide the state with assurance and allow it to return to “business as usual more quickly”. While this study stops short of evaluating the welfare impact of mandates, the results demonstrate the plausibility that mandates can be welfare increasing as the costs of reduced commerce may be offset by a faster return to more normal levels.

As other states around the country implement similar measures, including those proposed at the national level by the Biden administration for large businesses, Hawaii’s program can provide insight into potential outcomes. The positive impact on public health may influence others to follow suit. However, the resulting decrease in foot traffic must also be acknowledged by businesses. Measures can be taken to limit these losses. It should be noted that many businesses in the food and beverage industry chose to provide only takeout or delivery rather than dine-in options due to the mandates. This would imply decreased foot traffic was not necessarily decreasing revenue at the same rate, as some businesses may sell a similar amount of products for takeout or delivery that groups of customers would have previously eaten at the restaurant.

During the first week of the mandate’s implementation, the University of Hawaii Economic Research Organization (UHERO) surveyed 1,987 businesses throughout the state regarding their support of the mandates, expectations for how it would impact their businesses, and concerns [2]. Of businesses surveyed, 70.50% supported the mandates for employees to be vaccinated or regularly tested, and 61.75% supported the same for customers. The report estimates that the mandates led to an 8.00% increase in vaccination among employees compared with prior to the mandates. The main concerns cited among businesses included the ability to verify the vaccination or test status of both employees and customers.

As similar policies may be implemented in other states or Hawaii counties, this survey provides insight into how to combat potential business losses. Nearly half the businesses surveyed reported a need for technical assistance in verifying vaccination or test status, and more than one-third reported a need for further educational material for employees and customers [2]. Future use of such policies may find it beneficial to provide more information prior to implementation for both businesses and customers. Entities that implement COVID-19 mandates in the future may also wish to provide a way to verify vaccination and testing status beyond manually inspecting vaccination cards and test results.

Our study found that the vaccine or test passport program had a positive impact on public health in the state of Hawaii. Although this was encouraging in light of mixed results of other vaccination programs [18,19,20,21], the state of Hawaii is unique in its geographic location. Being an isolated island, the state has had much more autonomy in determining rules for entrance than mainland states. This evaluation cannot determine spillover effects that may be present in other states that implement similar policies. Some countries found similar effects on vaccination rates when requiring proof of vaccination or recent negative test or recovery for international travelers to enter or access public areas [15]. Additionally, the SafeGraph data used were limited to the individuals who agreed to location tracking and businesses in their panel. Finally, the vaccination data provided to the authors by the State of Hawaii Department of Health included only vaccines reported to the Vaccine Administration Management System and transferred to the Hawaii Immunization Registry, which does not include reports from many federal agencies. The non-reporting agencies include the military, which has a large presence in the state of Hawaii. While the results of this study show promising impacts of a vaccine mandate on public health, these limitations regarding both the data and extrapolation to other states or countries should be noted. The implications of these findings may be useful for determining future policies when taking the specific context of this mandate into account.

Finally, we note that the primary limitation of this model is the inability to formally prove that the parallel trends assumption is not violated [14]. While there is no formal test of this assumption, visually following the trend over time between the treatment and control in the pre-treatment period can be used to address this concern. Additionally, this model assumes there are no variables in either group that does not affect the other in the post-treatment period beyond the treatment itself [22]. An additional limitation of this model is finding two groups that meet these assumptions [23]. For our analysis, neither of these limitations should impact our results, as the counties appear to follow similar trends prior to treatment and there are no time-variant differences between the groups during this period that the authors can determine.

In conclusion, the COVID-19 vaccination or test passport program mandated in Honolulu and Maui provides evidence of a positive impact on public health and an initial loss for businesses. The significant decrease in both foot traffic (p<0.01, 95% Cl: 5.7–6.9%) and the COVID-19 case positivity rate (p<0.01, 95% Cl: 12.1–25.8%) and the significant increase in vaccination rate (p<0.05, 95% Cl: 0.29–2.55%) for impacted businesses and counties provides insight for future programs aimed at combating the pandemic. As these counties have been able to lift other restrictions following this program due to decreasing trends in cases and increasing trends in vaccination [1], businesses are likely to regain losses as they invest in the future safety of their communities. We note that our study focuses on the effects of mandates on businesses. The effects of mandates at schools and other venues, as well as for the use of public transportation, remain to be evaluated.

## Figures and Tables

**Table 1:** Summary statistics for the total sample and sub-sampled by the counties with and without vaccine mandates. Businesses in categories that fall under the mandates include those in the food and beverage, fitness, and entertainment industries. Statistics are for the entire sample period, before and after the mandates’ announcements and implementation. Estimates for businesses, COVID-19 cases, and county arrivals are estimated on a 7-day-rolling-average basis for each day, and vaccination rate increases are for each week. The observations column represents the total number of observations included in the analysis.

Variables	(1)	(2)	(3)	(4)	(5)
	Mean	St. Dev	Minimum	Maximum	Observations
**Total:**					
**7-day average daily business visits**	8.495	39.12	0	4257	3,266,485
**Businesses that fall under mandate**	0.287	0.452	0	1	3,267,000
**7-day average COVID-19 cases per 100,000 individuals**	25.35	20.44	2.830	89.47	1068
**Weekly vaccination rate increase**	0.686	0.287	0.250	1.523	68
**County Arrivals**	6048	4567	1174	18,380	1022
**Counties with Mandate (Honolulu & Maui):**					
**7-day average daily business visits**	9.508	44.79	0	4257	2,364,000
**Businesses that fall under mandate**	0.299	0.458	0	1	2,364,000
**7-day average COVID-19 cases per 100,000 individuals**	24.3	39.9	2.83	289	534
**Weekly vaccination rate increase**	0.766	0.271	0.0204	4.675	34
**County Arrivals**	9293	4098	2510	18,380	511
**Counties without Mandate (Hawaii and Kaui):**					
**7-day average daily business visits**	5.843	16.52	0	631.4	902,485
**Businesses that fall under mandate**	0.255	0.436	0	1	902,485
**7-day average COVID-19 cases per 100,000 individuals**	29.71	20.739	0	185	534
**Weekly vaccination rate increase**	0.607	0.284	0.250	5.898	34
**County Arrivals**	2408	827.9	581	4672	511

Estimates for 19,625 individual businesses from 1 July 21–30 December 21. COVID-19 case estimates and arrivals from 1 July 21–20 December 21. Vaccination rate increases from 17 June 21–11 October 21.

**Table 2:** Results for difference-in-difference regressions are described in Equations (5)–(8). The interaction terms estimate the impact of the mandates on businesses and counties that are impacted following announcement or implementation. These represent the change in the trend between impacted and non-impacted businesses or counties following the shock of the mandates, making these our coefficients of interest.

	ln(7-Day Average Daily Business Visits)				7-Day Average COVID-19 Cases Per 100,000			Weekly Dose-1 Vaccination Rate Increase					
								(all 18+)		(18–49)		(50+)	
Variables	(1)	(2)	(3)	(4)	(5)	(6)	(7)	(8)	(9)	(10)	(11)	(12)	(13)
					-	-	-	-	-	-	-	-	-
**Post-Mandate (9/13)**	−0.00911 ***	−0.00914 ***	−0.00766 ***	−0.00769 ***	−24.88 * ***		−25.79 ***	-	-	-	-	-	-
	(0.000580)	(0.00254)	(0.000593)	(0.00257)	(1.783)		(1.757)	-	-	-	-	-	-
**Businesses that fall under mandate**	0.359 ***		0.359 ***		-	-	-	-	-	-	-	-	-
	(0.0160)		(0.0157)		-	-	-	-	-	-	-	-	-
Post-mandate * under mandate	−0.0672 ***	−0.0672 ***	−0.0672 ***	−0.0672 ***	-	-	-	-	-	-	-	-	-
	(0.000791)	(0.00498)	(0.000791)	(0.00498)	-	-	-	-	-	-	-	-	-
**Counties with mandate**	-	-	-	-	73.93 ***	5.108	82.18 ***	−0.137	−0.953 ***	0.0480	−0.628 **	−0.185 ***	−0.325 ***
	-	-	-	-	(5.004)	(6.063)	(5.135)	(0.175)	(0.340)	(0.129)	(0.247)	(0.0570)	(0.116)
Post-Mandate * county with mandate	-	-	-	-	−9.599 ***	−6.630 ***	−9.955 ***	-	-	-	-	-	-
	-	-	-	-	(2.400)	(1.226)	(2.356)	-	-	-	-	-	-
**Post Mandate Announcement (8/5)**	-	-	-	-	-	-	-	−0.188 **		−0.144 **		−0.0438 *	
	-	-	-	-	-	-	-	(0.0749)		(0.0550)		(0.0243)	
Post-Announcement * county with mandate	-	-	-	-	-	-	-	0.130 *	0.157 **	0.0897 *	0.106 **	0.0398 *	0.0511 **
	-	-	-	-	-	-	-	(0.0721)	(0.0623)	(0.0530)	(0.0453)	(0.0234)	(0.0213)
**ln(New County COVID-19 Cases)**	−0.00147 ***	−0.00146 *	−0.00161 ***	−0.00160 *	-	-	-	-	-	-	-	-	-
	(0.000245)	(0.000773)	(0.000258)	(0.000863)	-	-	-	-	-	-	-	-	-
**ln(County Arrivals)**	0.134 ***	0.134 ***	0.143 ***	0.143 ***	−49.78 ***	−5.394	−55.10 ***	0.0380	0.518 **	−0.0467	0.355 **	0.0847 **	0.163 **
	(0.00103)	(0.00314)	(0.00107)	(0.00332)	−2.919	−3.849	−3.024	(0.102)	(0.211)	(0.0751)	(0.154)	(0.0332)	(0.0723)
**7-day COVID-19 test positivity rate**					-	-	-	0.107 ***	0.0338 *	0.0800 ***	0.0280 *	0.0266 ***	0.00575
					-	-	-	(0.0105)	(0.0197)	(0.00768)	(0.0143)	(0.00339)	(0.00676)
					-	-	-						
Observations	2,908,618	2,908,618	2,908,618	2,908,618	644	644	644	68	68	68	68	68	68
R-squared		0.019		0.019	0.471	0.244	0.495	0.771	0.732	0.771	0.720	0.716	0.682
Business fixed effects	No	Yes	No	Yes	-	-	-	-	-	-	-	-	-
Day-of-the-week fixed effects	No	No	Yes	Yes	No	No	Yes	-	-	-	-	-	-
County fixed effects	Yes	Yes	Yes	Yes	Yes	Yes	Yes	Yes	Yes	Yes	Yes	Yes	Yes
Day fixed effects	-	-	-	-	No	Yes	No	No	Yes	No	Yes	No	Yes
Number of Businesses	19,625	19,625	19,625	19,625	-	-	-	-	-	-	-	-	-
Number of Days	182	182	182	182	169	169	169	17	17	17	17	17	17

Standard errors reported in parentheses. Columns (1)–(4) regress variables onto the natural logarithm of daily visits to each individual business between 1 July 2021–31 December 2021. Businesses that fall under the vaccine or test passport mandate include those in the food and beverage industry, entertainment, and arts. Columns (5)–(7) regress variables onto the 7-day rolling average of new COVID-19 cases per 100,000 individuals for each county from 1 July 2021–20 December 2021. The counties that implemented the mandate are Honolulu and Maui. Foot traffic and COVID-19 test positivity observations are on a daily level. Columns (8)–(13) regress variables onto the increase in the county vaccination rates of the first dose administered weekly for all adults, those aged 18–49, and then for those 50+ from 17 June 2021–11 October 2021. Statistical significance denoted by *** *p* < 0.01, ** *p* < 0.05, * *p* < 0.1.

## Data Availability

All data is publicly available at [24].
